# Supplementary material for: Association between oral microbiome and seven types of cancers in East Asian population: a two-sample Mendelian randomization analysis
Source: Front Mol Biosci. 2023 Nov 21;10:1327893. doi: 10.3389/fmolb.2023.1327893 (PMC10702768; doi:10.3389/fmolb.2023.1327893)
Supplement: Supplementary file 1 [file DataSheet1.ZIP › Supplementary Materials/MR plots for tongue/tongue═╝/Gastric cancer/pheno.1960_to_gastric cancer_leave_one_out.pdf]

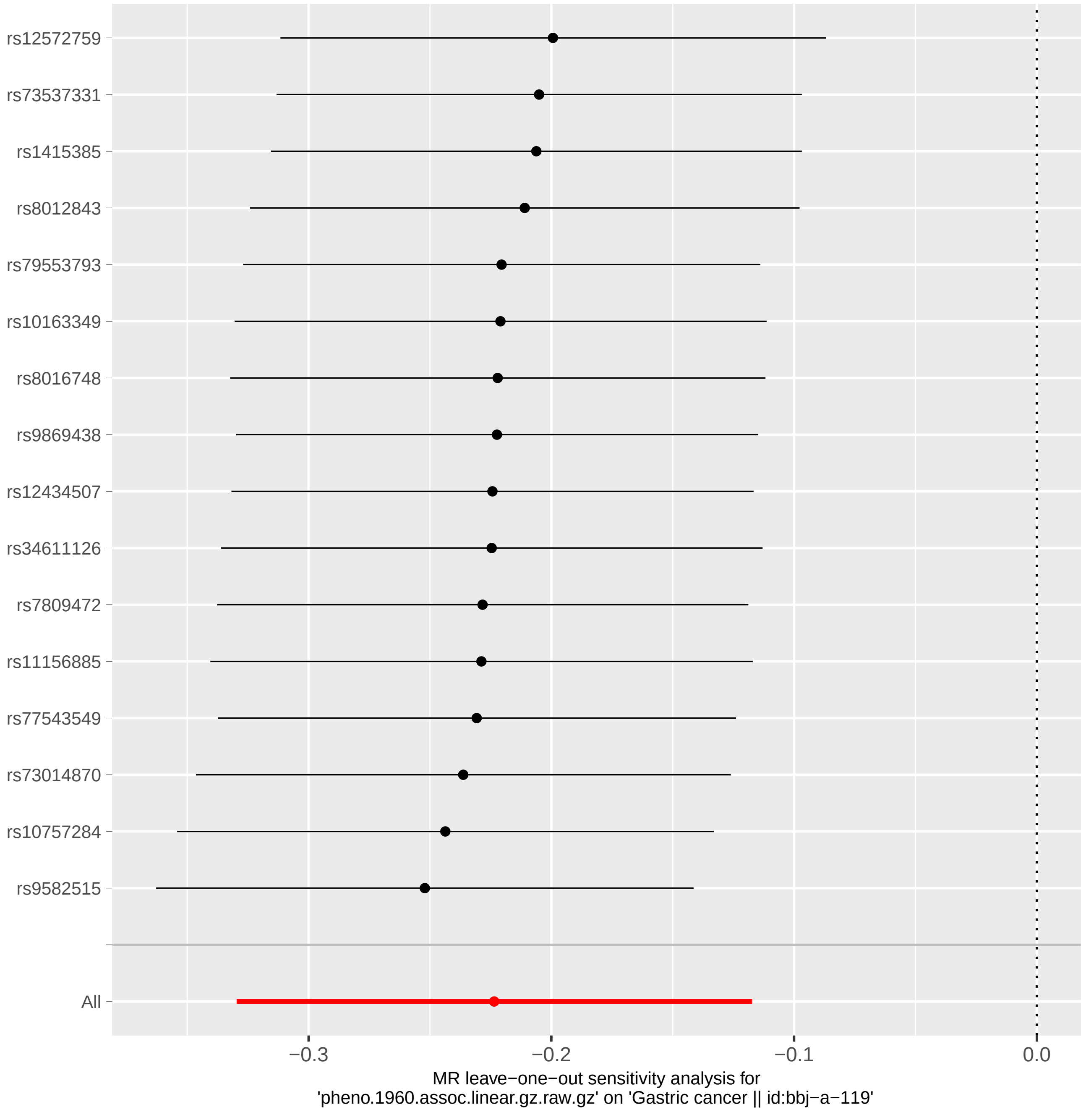
rs12572759
rs73537331
rs1415385
rs8012843
rs79553793
rs10163349
rs8016748
rs9869438
rs12434507
rs34611126
rs7809472
rs11156885
rs77543549
rs73014870
rs10757284
rs9582515
All
−0.3
−0.2
−0.1
0.0
MR leave−one−out sensitivity analysis for
'pheno.1960.assoc.linear.gz.raw.gz' on 'Gastric cancer || id:bbj−a−119'
